# Supplementary material for: Screening of Different Essential Oils Based on Their Physicochemical and Microbiological Properties to Preserve Red Fruits and Improve Their Shelf Life
Source: Foods. 2023 Jan 10;12(2):332. doi: 10.3390/foods12020332 (PMC9857945; doi:10.3390/foods12020332)
Supplement: Supplementary file 1 [file foods-12-00332-s001.zip › foods-2082182-supplementary.pdf]

foods

Supplementary Materials

Screening of Different Essential Oils Based on Their Physicochemical and Microbiological Properties to Preserve Red Fruits and Improve Their Shelf Life

Ziba Najmi [1], Alessandro Calogero Scalia [1], Elvira De Giglio [2,3], Stefania Cometa [4], Andrea Cochis [1], Antonio Colasanto [5], Monica Locatelli [5], Jean Daniel Coisson [5], Marcello Iriti [6,*], Lisa Vallone [7] and Lia Rimondini [1]

S1. FTIR/ATR analysis

The FT-IR spectra of the studied essential oils were reported in Figure S1.

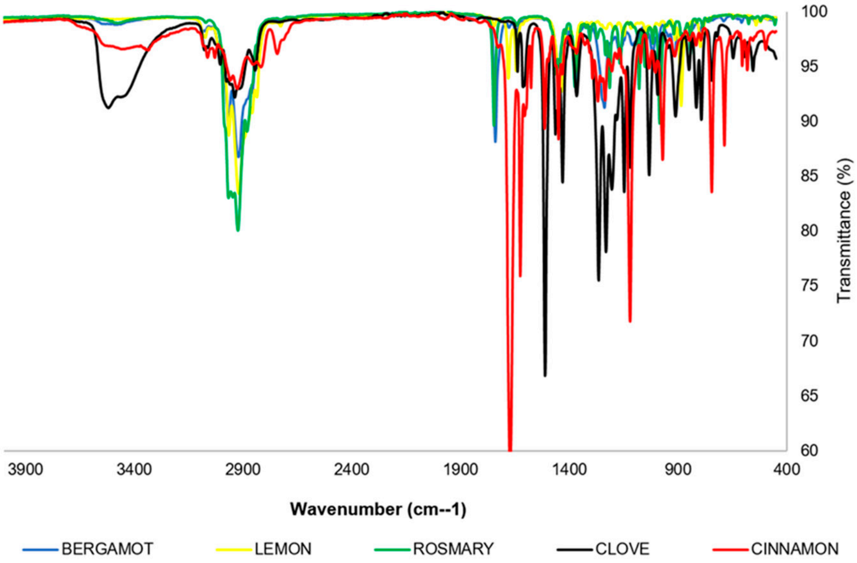

Supplementary Figure S1. FT-IR/ATR spectra of bergamot, lemon, rosemary, clove and cinnamon oils.

S2. Antibacterial activity of essential oils

Investigation of antibacterial activity of EOs was carried out against *E. coli* with disc diffusion test. Figure S2 shows inhibition halos of different concentrations of EOs.

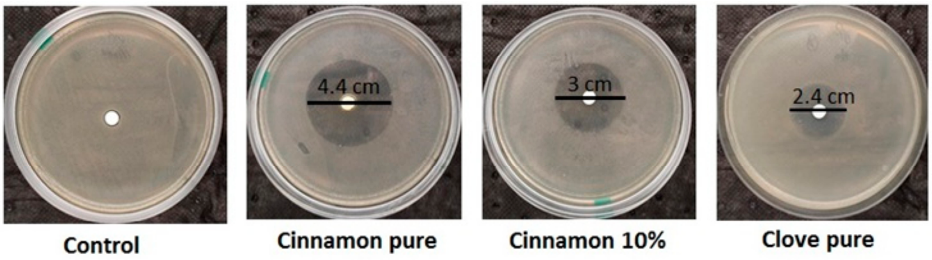

Supplementary Figure S2. Antibacterial activity of EOs against *E. coli*. 10% indicates the concentration (v/v) of EO; only positive results were presented.

Citation: Najmi, Z.; Scalia, A.C.; De Giglio, E.; Cometa, S.; Cochis, A.; Colasanto, A.; Locatelli, M.; Coisson, J.D.; Iriti, M.; Vallone, L.; et al. Screening of Different Essential Oils Based on Their Physicochemical and Microbiological Properties to Preserve Red Fruits and Improve Their Shelf Life. *Foods* **2023**, *12*, x. https://doi.org/10.3390/xxxxx

Academic Editor: Aurelio López-Malo

Received: 22 November 2022
Revised: 21 December 2022
Accepted: 4 January 2023
Published: 10 January 2023

Copyright: © 2023 by the authors. Submitted for possible open access publication under the terms and conditions of the Creative Commons Attribution (CC BY) license (https://creativecommons.org/licenses/by/4.0/).

S3. Assessment of Minimum Bactericidal Concentration (MBC)

In order to investigate MBC, LB agar plates were streaked with the same concentrations of EOs that were used for MIC assay. Figure S3 indicates images of LB agar plates indicating MBC.

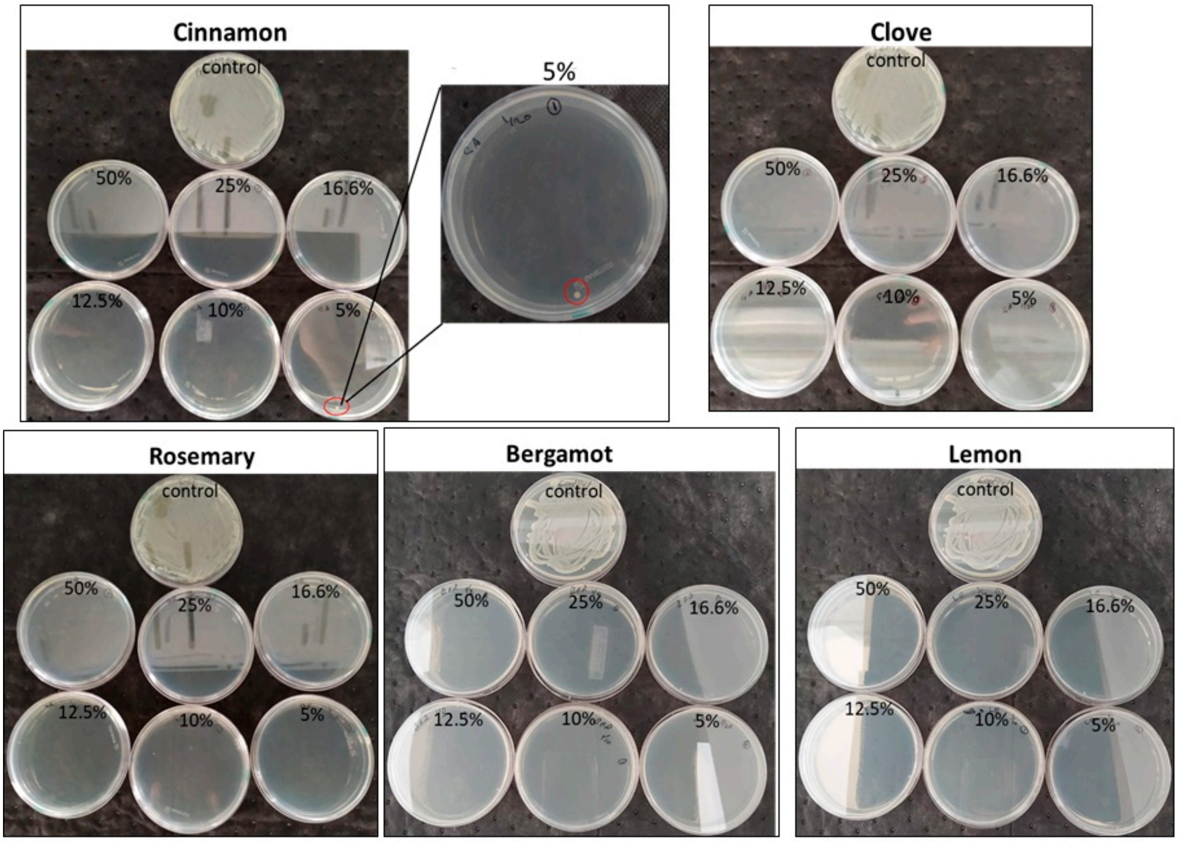

Supplementary Figure S3. Minimum Bactericidal Concentration (MBC) with a range of concentrations of EOs from 5% to 50% v/v.

S4. Antibacterial activity of binary combination of essential oils

Antibacterial activity of combined EOs was analyzed with disc diffusion against *E. coli* and after 24 hours, the diameter of inhibition halo was measured and compared with the diameter of inhibition halo of individual oil (single oil). Figure S4 a and b, show the comparison of inhibition halos between combined oils and single oil at concentration of 10% and 5% (v/v), respectively.

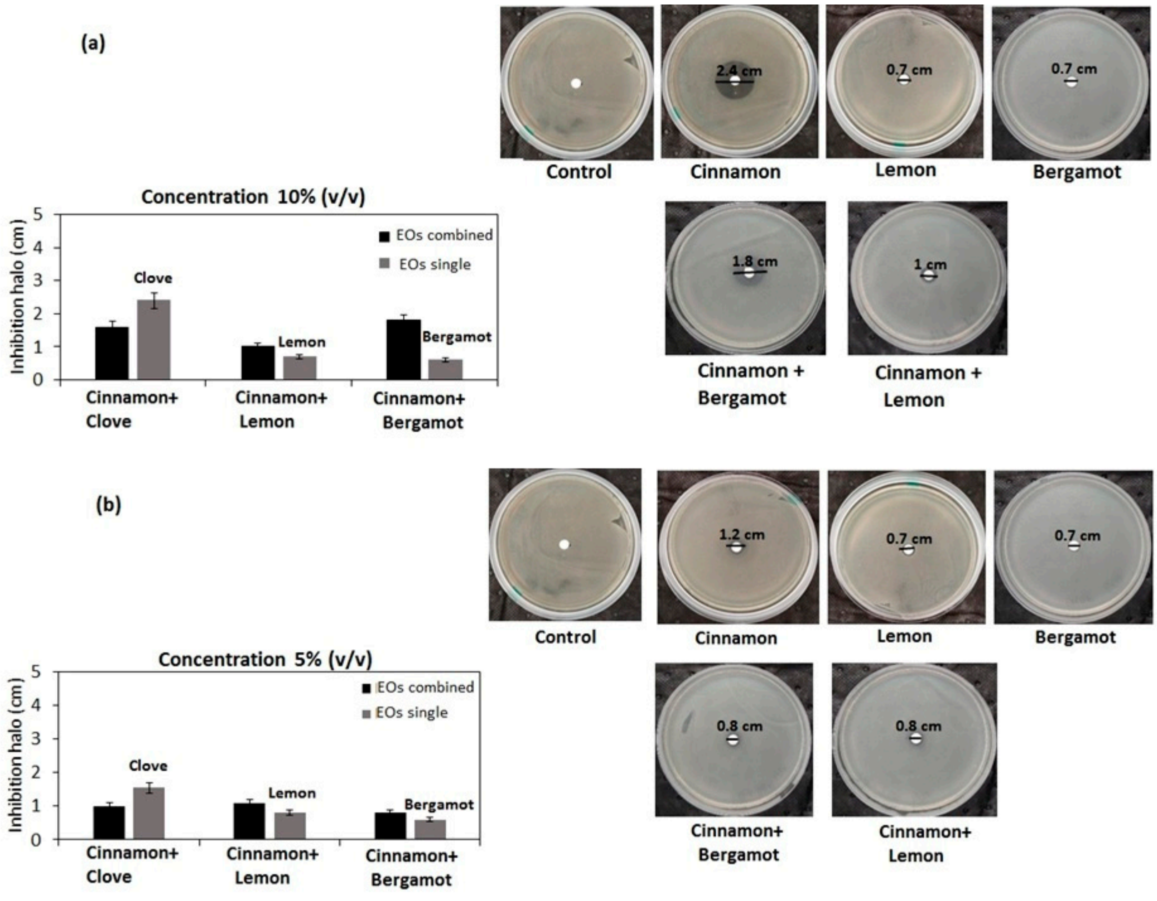

Supplementary Figure S4. Antibacterial activity of combined EOs against *E. coli*. (**a**) comparison of inhibition halos between combined oil and single oil (left panel) and pictures of petri dishes of bacterium (right panel) at concentration 10% (v/v); (**b**) comparison of inhibition halos between combined oil and single oil (left panel) and pictures of petri dishes of bacterium (right panel) at concentration 5% (v/v).
